# Healthcare Providers' Recommendations for Physical Activity among US Arthritis Population: A Cross-Sectional Analysis by Race/Ethnicity

**DOI:** 10.1155/2018/2807035

**Published:** 2018-02-04

**Authors:** Shamly Austin, Kenneth G. Saag, Maria Pisu

**Affiliations:** ^1^Research & Development, Gateway Health Plan®, Pittsburgh, PA 15222, USA; ^2^Division of Clinical Immunology and Rheumatology, Department of Medicine, University of Alabama at Birmingham, Birmingham, AL, USA; ^3^Division of Preventive Medicine, Department of Medicine, University of Alabama at Birmingham, Birmingham, AL, USA

## Abstract

**Introduction:**

We examined racial/ethnic disparities in healthcare providers' recommendations for physical activity among individuals with arthritis and evaluated this association among groups of individuals who adhered to physical activity guidelines and those who did not.

**Methods:**

With a cross-sectional design based on Behavioral Risk Factor Surveillance System, we analyzed individuals with self-reported physician-diagnosed arthritis, ≥18 years of age (*n* = 83,376). Outcome variable was healthcare providers' recommendations for physical activity. Race/ethnicity was categorized as African American, Hispanic, and White. Associations were examined using multivariate logistic regression.

**Results:**

African Americans (Adjusted OR: 0.66; 95% CI: 0.55–0.79) and Hispanics (Adjusted OR: 0.68; 95% CI: 0.56–0.83) were less likely to receive providers' recommendations.

**Conclusions:**

Although the importance of physical activity to improve health outcomes for adults with arthritis, as well as providers' influence on individuals' behavior change, is well established, providers are less likely to recommend physical activity to minorities. Further studies are required to identify the causes for this quality-of-care issue.

## 1. Introduction

According to 2013–2015 National Health Interview Survey, about 54.4 million US adults had self-reported physician-diagnosed arthritis, a number projected to reach more than 78.4 million by 2040 [1]. Prevalence, health impact, and economic consequences of arthritis are predicted to increase dramatically due to a growing aging population and obesity prevalence [2]. The 2008 Physical Activity Guidelines Advisory Committee on Musculoskeletal Health [3] recommends physical activity for the arthritis population, prescribing 150 minutes of light- to moderate-intensity physical activity per week to improve and maintain health-related quality of life. The 2012 American College of Rheumatology guidelines also recommend physical activity for the arthritis population [4].

Despite the known benefits of physical activity and current guidelines, adherence to physical activity guidelines is low in adults with arthritis (40–50%) [5, 6], particularly for racial/ethnic minorities. About 80% African American and 79% Hispanic adults were either inactive or did not meet the recommended levels of physical activity compared with 75% White adults [7]. One reason may be the lack of recommendations to be physically active from healthcare providers. Studies indicate that healthcare providers' recommendations act as catalyst towards individuals' health promoting behavior [6, 8, 9]. However, differences in recommendations by race/ethnicity are not uncommon [10–12]. Compared to Whites, minorities are less likely to receive guideline adherent care from their healthcare providers for mental health services [13, 14], kidney transplant procedures [15], cardiac procedures [16], or pain treatment [17].

It is currently not known if healthcare providers are less likely to recommend physical activity to minorities with arthritis and if observed disparities in physical activity are due to racial/ethnic differences in providers' recommendations. We address these knowledge gaps by examining data from 2011, 2013, and 2015 Behavioral Risk Factor Surveillance System Survey (BRFSS). Our objective was to determine the association between race/ethnicity and healthcare providers' recommendations for physical activity. We also examined this association among groups of individuals who adhered to physical activity guidelines and those who did not.

## 2. Materials and Methods

We conducted a retrospective cross-sectional study of individuals ≥18 years of age with self-reported physician-diagnosed arthritis based on 2011, 2013, and 2015 BRFSS survey data. The BRFSS is a random-digit-dial landline and cellular telephone household survey of the noninstitutionalized civilian US adult population, administered by the Centers for Disease Control and Prevention (CDC) [18]. Data for 17 states—California, Florida, Kansas, Kentucky, Michigan, Minnesota, Mississippi, Missouri, Montana, New York, Oregon, Pennsylvania, Rhode Island, South Carolina, Tennessee, Utah, and Wisconsin—were obtained. The outcome variable was the occurrence of a healthcare provider's recommendation for physical activity as obtained from BRFSS arthritis management module. This was assessed from responses to a question asking whether healthcare providers recommended physical activity or exercise to help with arthritis or joint symptoms (dichotomous variable: yes/no) [19, 20].

The predictor variable, race/ethnicity, was categorized as Whites, African Americans, and Hispanics.

To identify the variables that may confound the association between race/ethnicity and healthcare providers' recommendations for physical activity, we used the Social-Ecological Model. This model, used in several studies on physical activity [21–23], considers that individual, interpersonal, and social factors influence the individual's uptake of physical activity. Individual-level factors included in our analyses were age, gender, health insurance coverage, body mass index (BMI), health status, pain, number of comorbidities, and activity limitations. Age was measured as a continuous variable; health insurance coverage was measured as a dichotomous variable (have/do not have); BMI was categorized as obese, overweight, and underweight/normal; health status was measured as excellent, very good, good, fair, and poor based on how respondents considered their general health [19]. Pain was measured on a scale from 0 indicating no pain to 10 indicating aching pain as bad as it can be. The number of comorbidities was computed by summing up responses to questions on having or having been diagnosed with diabetes, myocardial infarction, angina, stroke, asthma, skin cancer, other cancers, chronic obstructive pulmonary disease, depressive disorder, and kidney diseases. The number of comorbidities ranged from 0 to 10. Activity limitations were measured from a question on whether arthritis or joint symptoms interfered with normal social activities, such as going shopping, going to the movies, or going to religious or social gatherings. Having activity limitations was measured as a dichotomous variable (yes/no).

Interpersonal-level factors were marital status (married versus other) and having a usual source of care (yes/no). Social-level factors included education (less than high school, high school, some college or technical education, and four-year college or higher), employment (employed, unemployed, and other (retired, homemakers, students, and disabled) as per the definitions from the Bureau of Labor Statistics) [24], annual income (less than $50,000 and greater than or equal to $50,000), and region of residence (northeast, midwest, south, and west). The above factors have been shown to affect providers' recommendations for physical activity [25–28] and adherence to physical activity guidelines [25] and vary across race/ethnicity. For example, compared with Whites, African Americans are more likely to be single [29], be unemployed [24], have lower income [30], have multiple chronic conditions [31], and reside in urban areas [32] and in Southern US [33]. Hispanics are more likely to have lower income [30], have multiple chronic conditions [31], and report activity limitations [34] compared to Whites.

*Analysis*. We summarized the descriptive characteristics for the overall sample by race/ethnicity. We conducted multivariate logistic regression models to examine the association between race/ethnicity and providers' recommendations adjusting for the individual, interpersonal, and social factors described above. Further, among individuals who adhered to the recommended guidelines of 150 minutes of physical activity per week and those who did not [20, 35], we conducted two separate logistic regressions to examine the association between race/ethnicity and providers' recommendations. Adherence to physical activity guidelines was a calculated BRFSS variable obtained from the physical activity module that recorded self-reported light, moderate, and vigorous physical activity in a usual week.

BRFSS uses a complex sampling design and CDC provides appropriate weights to adjust for survey noncoverage and nonresponses. We used the CDC provided weights to weight the sample to the US population [36]. Data management and analyses were conducted using SPSS version 23 (IBM, Armonk, NY). We used SPSS Complex Samples to account for BRFSS Complex Samples methodology [36]. Data for the study were obtained from de-identified publically available datasets.

## 3. Results

Our sample included 83,376 individuals with self-reported arthritis corresponding to a weighted sample of 51.5 million. Overall, 80% were Whites, 9.7% African Americans, and 10.0% Hispanic individuals (Table 1). About 74.4% were obese or overweight, the average level of pain was five, and the number of comorbidities was one. About 33% had fair to poor health status, 48% reported activity limitations, 15% did not have any usual source of care, 35% were employed, and 55% had income <$50,000.

Across the different race/ethnicity groups, more African Americans (55%) and Hispanics (46%) than Whites (37%) were obese. Higher rates of poor health were reported by African Americans (44%) and Hispanics (52%) compared with Whites (30%). In addition, 21% of Hispanics did not have a usual source of care compared with 18% African Americans and 15% Whites. About 69% African Americans and Hispanics had an annual income of <$50,000 compared with 52% of Whites, and 40% Hispanics and 24% African Americans had education level less than high school compared with 12% Whites (Table 1).

In the overall sample (*n*  =  83,376), 58.2% received a recommendation for physical activity and among them 48.1% adhered to physical activity guidelines. Among those who received providers' recommendations, adherence was 36%, 41%, and 52.4% for African Americans, Hispanics, and Whites, respectively (Table 2).

Results from the logistic regression model, after adjusting for individual, interpersonal, and social factors, found that African Americans (Adjusted OR: 0.66; 95% CI: 0.54–0.78) and Hispanics (Adjusted OR: 0.68; 95% CI: 0.56–0.83) were less likely to receive providers' recommendations compared with Whites (Table 3). In the overall sample, being female (Adjusted OR: 0.74; 95% CI: 0.69–0.81), obese (Adjusted OR: 0.57; 95% CI: 0.52–0.63), or overweight (Adjusted OR: 0.79; 95% CI: 0.72–0.87), health status reported as very good (Adjusted OR: 0.84; 95% CI: 0.72–0.98) or good (Adjusted OR: 0.78; 95% CI: 0.67–0.91), and activity limitations (Adjusted OR: 0.74; 95% CI: 0.68–0.80) were factors associated with a lower likelihood of receiving providers' recommendations (Table 3). Further, having education less than four-year college (Adjusted OR: 1.71; 95% CI: 1.47–1.98), no health coverage (Adjusted OR: 1.39; 95% CI: 1.15–1.67), and no usual source of care (Adjusted OR: 1.29; 95% CI: 1.15–1.44) were factors associated with a higher likelihood of receiving providers' recommendations (Table 3). Across all three racial/ethnic groups, the common factors associated with the likelihood of receiving recommendations were education and pain level (Table 3).

Overall, 40,743 (49%) individuals adhered to physical activity guidelines, and 42,633 (51%) did not adhere. Among those who did not adhere to physical activity guidelines, 57.3% had a provider's recommendation for physical activity (69.3% of African Americans, 68.1% of Hispanics, and 55.4% of Whites) (Table 2). After adjusting for covariates in the logistic regression model, we found that, among those who did not adhere to physical activity guidelines, African Americans (Adjusted OR: 0.55; 95% CI: 0.43–0.70) and Hispanics (Adjusted OR: 0.56; 95% CI: 0.44–0.72) were less likely to receive providers' recommendations for physical activity than Whites. Among those who adhered to physical activity guidelines, no significant association was found between race/ethnicity and receiving a recommendation (Table 4).

## 4. Discussion

In a sample of BRFSS respondents representative of the US adult population with arthritis, we observed that 6 in 10 individuals reported receiving recommendations for physical activity from their healthcare providers, and African Americans and Hispanics were less likely to receive providers' recommendations for physical activity compared to Whites. These differences occurred among respondents who were not sufficiently active, and not among those who were physically active and adhered to current guidelines for physical activity. Moreover, we found that the group of respondents with additional health conditions (obese and overweight, those with higher pain levels, comorbidities, and activity limitations) who are most in need for these recommendations were less likely to receive them.

About 150 minutes of light- to moderate-intensity physical activity per week [4] is recommended for individuals with arthritis to improve and maintain health-related quality of life [37]. Yet, healthcare providers do not follow the recommended guidelines for physical activity; reasons may be related to knowledge-, attitude-, and behavior-related barriers [6, 25, 38]. Knowledge-related barriers include lack of familiarity or awareness of the guidelines; attitude-related barriers include lack of agreement with guidelines, self-efficacy, and outcome expectancy; and behavior-related barriers include lack of time, resources, reimbursements, organizational constraints, or perceived increase in malpractice liability [6, 25, 38]. Given the established importance of physical activity to improve outcomes for adults with arthritis [4, 5], it is fundamental to understand if lack of providers' recommendations for physical activity is indeed a quality-of-care problem. Previous studies show that a recommendation to be physically active from healthcare providers is positively associated with individuals' acceptance of health promoting behavior [6, 8, 9]. However, unconscious stereotypes [10–12], especially related to the ability of individuals to exercise, may affect the degree to which healthcare providers recommend physical activity to minorities. Moreover, among individuals who are not physically active, providers' recommendations alone may not be enough to take up physical activity. There might be other factors such as nonavailability of open spaces, lack of access to gymnasiums, neighborhood crime rate, and multiple jobs that may prevent adherence to physical activity in racial/ethnic minorities [39].

Pain, stiffness, fatigue, and disability are reported as frequent barriers to physical activity [40]. Although physical activity has been shown to improve these symptoms [37, 40] and should be recommended irrespective of disease severity, pain levels, and functional status [4, 41], our results show that individuals who are in need and are most likely to benefit (obese/overweight, those with higher pain levels, comorbidities, and activity limitations) were less likely to receive healthcare providers' recommendations. Moderate-intensity aerobic exercises such as brisk walking as well as vigorous-intensity aerobics such as jogging and swimming, muscle strengthening exercises such as gardening, working with resistance bands, and balance activities such as Tai Chi and flexibility exercises are some of the types of activities recommended for the arthritis population [37]. Individuals should find out the activity that best works for them in consultation with their healthcare providers [37]. Physical inactivity worsens balance, pain tolerance, and joint stiffness and weakens muscles [42] and, thus, affects individuals' current as well as future health-related quality of life. Hence, it is important to identify strategies to improve adherence to physical activity guidelines in people with arthritis, especially in those who have symptoms and limitations.

Among individuals (of all race/ethnicity groups) who adhered to physical activity guidelines, we did not find an association between healthcare providers' recommendations for physical activity and race/ethnicity. It might be that these individuals are self-motivated and do not need their providers' recommendations to meet the recommended guidelines. However, among individuals who did not adhere to physical activity guidelines, we observed African Americans and Hispanics being less likely to receive recommendations for physical activity from their providers. Studies have shown that providers' recommendations for physical activity translate into behavior change among individuals [8, 43]. The Physician-Based Assessment and Counseling for Exercise (PACE) project concluded that short provider counseling sessions of three to five minutes increased physical activity [42]. More than 50% of the providers believed that after the brief counseling their patients did increase their level of physical activity [43]. Thus, providers can play a pivotal role as educators and motivators in changing individuals' behavior especially among minorities who are less engaged in physical activity.

Our results should be considered in the light of certain limitations. The survey excludes households without telephone or cell phone and individuals in institutions and the military. Hence, results may not be generalizable to these populations. BRFSS responses are self-reported and not obtained from medical reports. Individuals who had a recent visit to their healthcare provider may recall better their provider visit than those who visited a few months earlier; hence, there is a possibility of recall bias or social desirability bias. The association between providers' recommendations and race/ethnicity may be confounded by variables such as stiffness, fatigue, individuals' past exercise behavior, availability of time, and neighborhood crime rate. In addition, we do not have information on the content of the conversations between healthcare providers and individuals with arthritis, which may shed light on nonadherence in African Americans despite receiving recommendations for physical activity.

## 5. Conclusion

Despite the limitations, this is the first study based on a large population-based survey that analyzes racial/ethnic disparities in healthcare providers' recommendations for physical activity and examines this association among individuals who were adherent or not adherent to physical activity. Our study implies that, for individuals with arthritis to maintain an active lifestyle, provider-level recommendations alone may not be enough. Recommendations should be supported by health education and inbuilt environment for physical activity. Future research should focus on provider–patient communication and adherence to physical activity guidelines and evaluate how providers' recommendations for physical activity are perceived by different racial/ethnic groups.

## Figures and Tables

**Table 1:** Characteristics of individuals with self-reported arthritis from 2011, 2013, and 2015 Behavioral Risk Factor Surveillance System Survey.

Variables	Overall (*n* = 83,376) (%)	White (*n* = 73,507) (%)	African American (*n* = 8,005) (%)	Hispanic (*n* = 1,864) (%)	*p* value
*Individual factors*					
Age (in years)					
Mean (±SD)	59.6 (±14.5)	60.5 (±14.4)	57.3 (±14.4)	54.8 (±14.1)	0.000^*∗*^
Age (groups)					
18–44 years	15.1	13.9	18.4	21.8	0.000^*∗*^
45–64 years	46.0	44.6	49.6	53.2	
≥65 years	38.9	41.5	31.9	25.0	
Gender					
Female	58.3	57.5	57.7	65.4	0.001^*∗*^
Race					
White	80.3	—	—	—	
African American	9.7	—	—	—	
Hispanic of any race	10.1	—	—	—	
Health coverage					
Yes	91.7	92.8	86.2	88.0	0.000^*∗*^
Body mass index					
Obese	39.9	37.3	54.5	46.3	0.000^*∗*^
Overweight	34.5	35.2	28.1	35.2	
Normal/underweight	25.6	27.5	17.4	18.6	
Health status					
Excellent	7.9	8.5	4.3	6.9	0.000^*∗*^
Very good	25.6	28.0	17.4	14.4	
Good	33.1	33.8	34.0	27.1	
Fair	22.1	19.4	30.6	35.3	
Poor	11.3	10.4	13.7	16.3	
Pain					
Median (IQR)	5 (3,7)	5 (2,7)	6 (4,8)	6 (3,8)	0.000^*∗*^
Number of comorbidities					
Median (IQR)	1 (0,2)	1 (0,2)	1 (0,2)	1 (0,2)	0.365
Activity limitation					
No	52.3	51.7	53.4	56.7	0.072
Yes	47.7	48.3	46.6	43.3	
*Interpersonal factors*					
Marital status					
Married	45.1	42.4	64.6	47.1	0.000^*∗*^
Usual source for care					
Yes	84.6	85.5	82.5	79.1	0.001^*∗*^
*Social factors*					
Education					
≤High school	16.2	12.4	23.7	39.5	0.000^*∗*^
High school	30.5	31.7	30.1	20.9	
Some college or technical education	33.1	33.6	33.0	28.9	
4-year college or higher	20.3	22.3	13.2	10.7	
Employment					
Unemployed	6.3	5.7	9.4	8.7	0.003^*∗*^
Others (retired, homemakers, disabled, students)	58.4	58.7	57.4	56.8	
Employed	35.3	35.6	33.3	34.5	
Income					
<$50,000	55.3	51.9	69.1	68.8	0.000^*∗*^
≥$50,000	33.1	35.7	19.0	25.2	
Undisclosed	11.7	12.4	11.8	6.0	
Region					
Northeast	17.5	17.6	20.0	14.4	0.000^*∗*^
Midwest	31.4	34.8	27.6	7.4	
South	19.9	20.0	30.5	8.2	
West	31.2	27.5	21.9	70.0	

Sample weighted to the US population;  ^*∗*^significant *p* < 0.05.

**Table 2:** Healthcare providers' recommendations for physical activity and individuals' adherence to physical activity guidelines in the overall sample and among those who adhered to recommended guidelines and those who did not.

			Adhered to physical activity guidelines (*n* = 40,743)	Did not adhere to physical activity guidelines (*n* = 42,633)
Sample	*Overall* providers' recommendations for physical activity (*n* = 83,376) (%)	Individuals who received recommendations and adhered to physical activity guidelines (*n* = 47,296) (%)	Individuals who received providers' recommendations (*n* = 23,445) (%)	Individuals who received providers' recommendations (*n* = 24,481) (%)

Whole sample	58.2	48.1	58.0	58.5
African American	67.1	36.0	63.4	69.3
Hispanic	64.7	40.8	60.2	68.1
White	56.4	52.4	57.3	55.4

Sample weighted to the US population.

**Table 3:** Logistic regression showing the association between healthcare providers' recommendations for physical activity and race/ethnicity among adults with arthritis (BRFSS 2011, 2013, and 2015).

Variables (reference)	Measures	Overall (*n* = 83,376)	African American (*n* = 8,005)	Hispanic (*n* = 1,864)	White (*n* = 73,507)
		Adjusted Odds Ratio (95% CI)	Adjusted Odds Ratio (95% CI)	Adjusted Odds Ratio (95% CI)	Adjusted Odds Ratio (95% CI)
*Individual factors*					
Race (White)	African American	0.66 (0.54–0.78)_ _^*∗*^	—	—	—
	Hispanic of any race	0.68 (0.56–0.83)_ _^*∗*^	—	—	—
Age (in years)		1.00 (1.00–1.01)	0.99 (0.98–1.01)	0.99 (0.98–1.01)	1.00 (1.00–1.01)
Gender (male)	Female	0.74 (0.69–0.81)_ _^*∗*^	0.72 (0.54–0.97)_ _^*∗*^	0.72 (0.49–1.05)	0.75 (0.69–0.80)
Health coverage (yes)	No	1.39 (1.15–1.67)_ _^*∗*^	1.75 (1.07–2.86)_ _^*∗*^	0.87 (0.50–1.54)	1.38 (1.16–1.64)_ _^*∗*^
Body mass index (normal/underweight)	Obese	0.57 (0.52–0.63)_ _^*∗*^	0.59 (0.41–0.83)_ _^*∗*^	0.63 (0.40–1.00)	0.56 (0.50–0.60)_ _^*∗*^
	Overweight	0.79 (0.72–0.87)_ _^*∗*^	0.60 (0.42–0.86)_ _^*∗*^	0.91 (0.55–1.47)	0.79 (0.72–0.87)_ _^*∗*^
Health status (excellent)	Very good	0.84 (0.72–0.98)_ _^*∗*^	1.05 (0.55–2.01)	0.75 (0.33–1.71)	0.83 (0.71–0.97)_ _^*∗*^
	Good	0.78 (0.67–0.91)_ _^*∗*^	0.73 (0.40–1.32)	0.91 (0.41–2.03)	0.77 (0.66–0.89)_ _^*∗*^
	Fair	0.89 (0.74–1.06)	1.06 (0.56–2.01)	0.88 (0.38–1.99)	0.87 (0.73–1.03)
	Poor	1.05 (0.86–1.28)	1.36 (1.69–2.67)	1.05 (0.40–2.76)	1.03 (0.84–1.25)
Pain		0.91 (0.89–0.92)_ _^*∗*^	0.89 (0.84–0.94)_ _^*∗*^	0.90 (0.83–0.97)_ _^*∗*^	0.91 (0.90–0.93)_ _^*∗*^
Number of comorbidities		0.95 (0.92–0.98)_ _^*∗*^	0.84 (0.75–0.95)_ _^*∗*^	0.92 (0.80–1.06)	0.96 (0.94–0.99)_ _^*∗*^
Activity limitations (no)	Yes	0.74 (0.68–0.80)_ _^*∗*^	0.88 (0.65–1.17)	0.55 (0.37–0.81)_ _^*∗*^	0.75 (0.68–0.81)_ _^*∗*^
*Interpersonal factors*					
Marital status (married)	Others	1.15 (1.05–1.25)_ _^*∗*^	1.18 (0.85–1.65)	1.42 (0.94–2.14)	1.11 (1.02–1.20)_ _^*∗*^
Usual source for care (yes)	No	1.29 (1.15–1.44)_ _^*∗*^	1.34 (0.94–1.92)	1.47 (0.92–2.35)	1.23 (1.11–1.37)_ _^*∗*^
*Social factors*					
Education (4-year college or higher)	<High school	1.71 (1.47–1.98)_ _^*∗*^	1.77 (1.14–2.74)_ _^*∗*^	2.91 (1.55–5.46)_ _^*∗*^	1.65 (1.41–1.92)_ _^*∗*^
	High school	1.35 (1.22–1.450)_ _^*∗*^	1.34 (0.90–1.98)	1.95 (1.04–3.67)_ _^*∗*^	1.33 (1.20–1.48)_ _^*∗*^
	Some college or technical education	1.14 (1.03–1.26)_ _^*∗*^	1.13 (0.73–1.72)	2.12 (1.19–3.76)_ _^*∗*^	1.10 (0.99–1.20)
Employment (employed)	Unemployed	0.98 (0.80–1.21)	0.95 (0.51–1.77)	0.60 (0.26–1.40)	1.06 (0.88–1.29)
	Others (retired, homemakers, disabled, students)	0.93 (0.84–1.03)	0.93 (0.64–1.35)	0.67 (0.42–1.06)	0.97 (0.87–1.07)
Income (≥$50,000)	<$50,000	1.15 (1.02–1.29)_ _^*∗*^	0.71 (0.41–1.23)	1.52 (0.91–2.56)	1.15 (1.04–1.27)_ _^*∗*^
	Income undisclosed	1.16 (1.00–1.32)	0.75 (0.39–1.46)	1.83 (0.92–3.61)	1.16 (1.02–1.31)_ _^*∗*^
Region (west)	Northeast	0.96 (0.84–1.08)	0.81 (0.48–1.38)	0.82 (0.52–1.29)	1.00 (0.88–1.14)
	Midwest	1.02 (0.91–1.15)	0.82 (0.49–1.36)	1.08 (0.69–1.67)	1.07 (0.96–1.19)
	South	1.03 (0.91–1.16)	0.81 (0.48–1.38)	0.86 (0.51–1.46)	1.08 (0.96–1.22)

Samples were weighted to the US population;  ^*∗*^significant *p* < 0.05.

**Table 4:** Logistic regression showing the association between race/ethnicity and providers' recommendations for physical activity among individuals who adhered to physical activity guidelines and those who did not adhere.

Variables (reference category)	Measures	Adhered to physical activity (*n* = 40,743)	Did not adhere to physical activity (*n* = 42,633)
		Adjusted Odds Ratio (95% CI)	Adjusted Odds Ratio (95% CI)
*Individual factors*			
Race (White)	African American	0.83 (0.64–1.07)	0.55 (0.43–0.70)_ _^*∗*^
	Hispanic of any race	0.85 (0.63–1.16)	0.56 (0.44–0.72)_ _^*∗*^
Age (in years)		1.01 (1.00–1.01)	1.00 (0.99–1.01)
Gender (male)	Female	0.67 (0.60–0.76)_ _^*∗*^	0.81 (0.73–0.90)_ _^*∗*^
Health coverage (yes)	No	1.24 (0.96–1.57)	1.49 (1.14–1.94)_ _^*∗*^
Body mass index (normal/underweight)	Obese	0.55 (0.47–0.62)_ _^*∗*^	0.56 (0.49–0.64)_ _^*∗*^
	Overweight	0.79 (0.68–0.89)_ _^*∗*^	0.78 (0.68–0.88)_ _^*∗*^
Health status (excellent)	Very good	0.85 (0.70–1.02)	0.86 (0.66–1.13)
	Good	0.77 (0.64–0.93)_ _^*∗*^	0.83 (0.64–1.08)
	Fair	0.81 (0.63–1.03)	0.95 (0.72–1.27)
	Poor	0.79 (0.57–1.10)	1.14 (0.85–1.53)
Pain		0.88 (0.86–0.91)_ _^*∗*^	0.92 (0.90–0.94)_ _^*∗*^
Number of comorbidities		0.94 (0.90–0.98)_ _^*∗*^	0.96 (0.92–1.00)
Activity limitations (no)	Yes	0.70 (0.63–0.79)_ _^*∗*^	0.74 (0.67–0.84)_ _^*∗*^
*Interpersonal factors*			
Marital status (married)	Others	1.19 (1.05–1.35)_ _^*∗*^	1.11 (0.99–1.25)
Usual source for care (yes)	No	1.28 (1.09–1.50)_ _^*∗*^	1.32 (1.13–1.54)_ _^*∗*^
*Social factors*			
Education (4-year college or higher)	<High school	1.69 (1.31–2.13)_ _^*∗*^	1.72 (1.45–2.06)_ _^*∗*^
	High school	1.31 (1.13–1.52)_ _^*∗*^	1.39 (1.21–1.59)_ _^*∗*^
	Some college or technical education	1.09 (0.95–1.25)	1.21 (1.04–1.39)_ _^*∗*^
Employment (employed)	Unemployed	1.02 (0.76–1.37)	0.98 (0.74–1.28)
	Others (retired, homemakers, disabled, students)	0.87 (0.75–1.00)	1.01 (0.86–1.15)
Income (≥$50,000)	<$50,000	1.16 (0.99–1.35)	1.12 (0.94–1.33)
	Income undisclosed	1.22 (1.02–1.47)	1.10 (0.90–1.33)
Region (west)	Northeast	0.85 (0.71–1.01)	1.05 (0.86–1.26)
	Midwest	0.98 (0.85–1.14)	1.08 (0.90–1.28)
	South	0.92 (0.78–1.08)	1.09 (0.90–1.31)

Samples were weighted to the US population;  ^*∗*^significant *p* < 0.05.
